# Construction of a cell-based aggregation and seeding model for the Tau protein: A cell-based aggregation and seeding model for the Tau protein

**DOI:** 10.3724/abbs.2024057

**Published:** 2024-04-29

**Authors:** Jiying Hu, Liqiang Wang, Jie Chen, Yi Liang

**Affiliations:** 1 Hubei Key Laboratory of Cell Homeostasis College of Life Sciences TaiKang Center for Life and Medical Sciences Wuhan University Wuhan 430072 China; 2 Office of Core Facility Shenzhen Bay Laboratory Shenzhen 518000 China

A pathological hallmark of Alzheimer’s disease (AD), the most common neurodegenerative disease in elderly people, is the formation of neurofibrillary tangles (NFTs), which are mainly composed of bundles of amyloid fibrils formed by abnormal deposition of hyperphosphorylated full-length human Tau protein [ 1– 3] . Recent studies have shown that AD-related cognitive decline and brain atrophy are closely correlated with Tau PET signal, further supporting the link between Tau pathology and AD symptomatology [ 4, 5] . Despite the high incidence and severe burden to patients, caregivers, and health systems caused by AD, there are few disease-modifying therapies available.

Normal functional human Tau protein binds to tubulin heterodimers through its microtubule-binding repeats and stabilizes microtubules. Hyperphosphorylated Tau detaches from microtubules and exposes the microtubule-binding domain, thereby leading to Tau self-oligomerization and aggregation [6]. Accumulating evidence suggests that filamentous Tau inclusions form first in a small number of brain cells, from which they are released and taken up by neighboring cells via endocytosis; these filamentous Tau inclusions act as templates for their own replication through monomeric Tau addition and propagate to other regions of the cells [ 7, 8] . Propagation of neuropathology is called prion-like, which refers to the capacity of an abnormally assembled protein to induce the same pathological conformation in the same protein, initiating a self-amplifying cascade. Transcellular propagation and prion-like phenomena are thought to contribute to the progression of pathology in AD, suggesting that inhibiting Tau aggregation and seeding could slow disease progression [ 7, 8] .

Accordingly, different experimental aggregation models for the Tau protein have been developed. A cell-based model offers a physiological assay environment with controllable costs and reproducible results, making it the most widely used model for the development of Tau-targeted therapies. In most cellular models, self-assembly of naive monomeric Tau is promoted by the addition of an exogenous ‘‘seed’’ template of synthetic or patient-derived pre-aggregated Tau [9]. Pre-prepared fibrillar seeds of Tau are added to the cell culture medium, taken up by cells, and act as templates to induce the aggregation of monomeric Tau. In addition to homotypic seeding, heterotypic seeding has also been demonstrated for Tau. Direct cross-seeding between pre-aggregated Aβ and Tau is supported by direct binding between Aβ peptides and Tau and direct induction of Tau fibrillization by pre-aggregated Aβ seeds [10]. Human-derived seeds are the most relevant source of pathological Tau protein; however, clinical material is not straightforward to obtain and work with, and it is difficult to guarantee the quality and stability of aggregated seeds. In addition, aggregate seeds could damage the integrity of the cell membrane and lead to cytotoxicity.

In the present study, we reported the construction of a cell-based model for the aggregation of endogenous Tau protein in cells without pre-prepared seeds. By introducing an aggregation-driven pathological mutant, ΔK280, to the aggregation-prone truncated core fragment of Tau (Tau _244‒372_, K18), we constructed a stable cell line over-expressing K18-ΔK280. Over-expressed K18-ΔK280 spontaneously aggregated in SH-SY5Y cells, forming amyloid fibrils positive for thioflavin S (ThS) ( Figure 1), a fluorescent dye with β-sheet binding properties, which is widely employed to observe amyloid plaque accumulation [10]. Based on the present cellular model, the properties of Tau aggregation after seeding can be further observed. The aggregates formed by K18-ΔK280 induce co-aggregation and phosphorylation of endogenous Tau in SH-SY5Y cells, which can be recognized by AT8 (phosphorylation at Ser202/Thr205) and pS396 (phosphorylation at Ser396) ( Figures 2 and 3) because phosphorylation at Ser202, Thr205, and Ser396 occurred in endogenous Tau but not at K18-ΔK280. This model is easy to use and avoids the potential cytotoxicity caused by fibrillar seeds.

**Figure 1:**
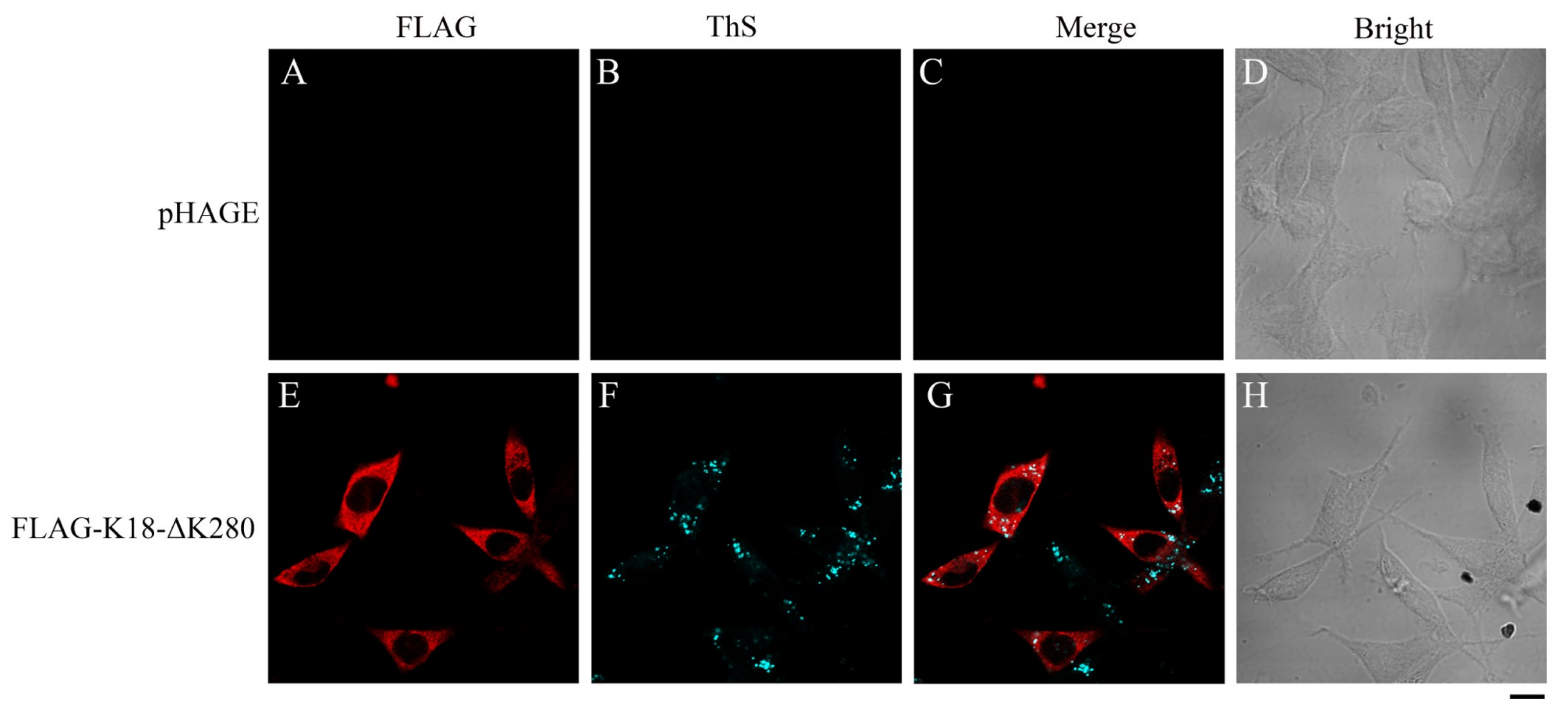
Figure 1 Aggregation of K18-ΔK280 in stable SH-SY5Y cells detected by ThS staining SH-SY5Y cells stably transfected with empty pHAGE plasmid (A–D) and FLAG-tagged K18-ΔK280 (E–H) were fixed with paraformaldehyde, permeabilized with 0.25% Triton X-100, stained with 0.1% ThS (cyan), sequentially coimmunostained with primary monoclonal anti-FLAG antibody (red) and secondary Alexa Fluor 546-conjugated IgG, and visualized by confocal microscopy. Scale bar: 10 μm.

**Figure 2:**
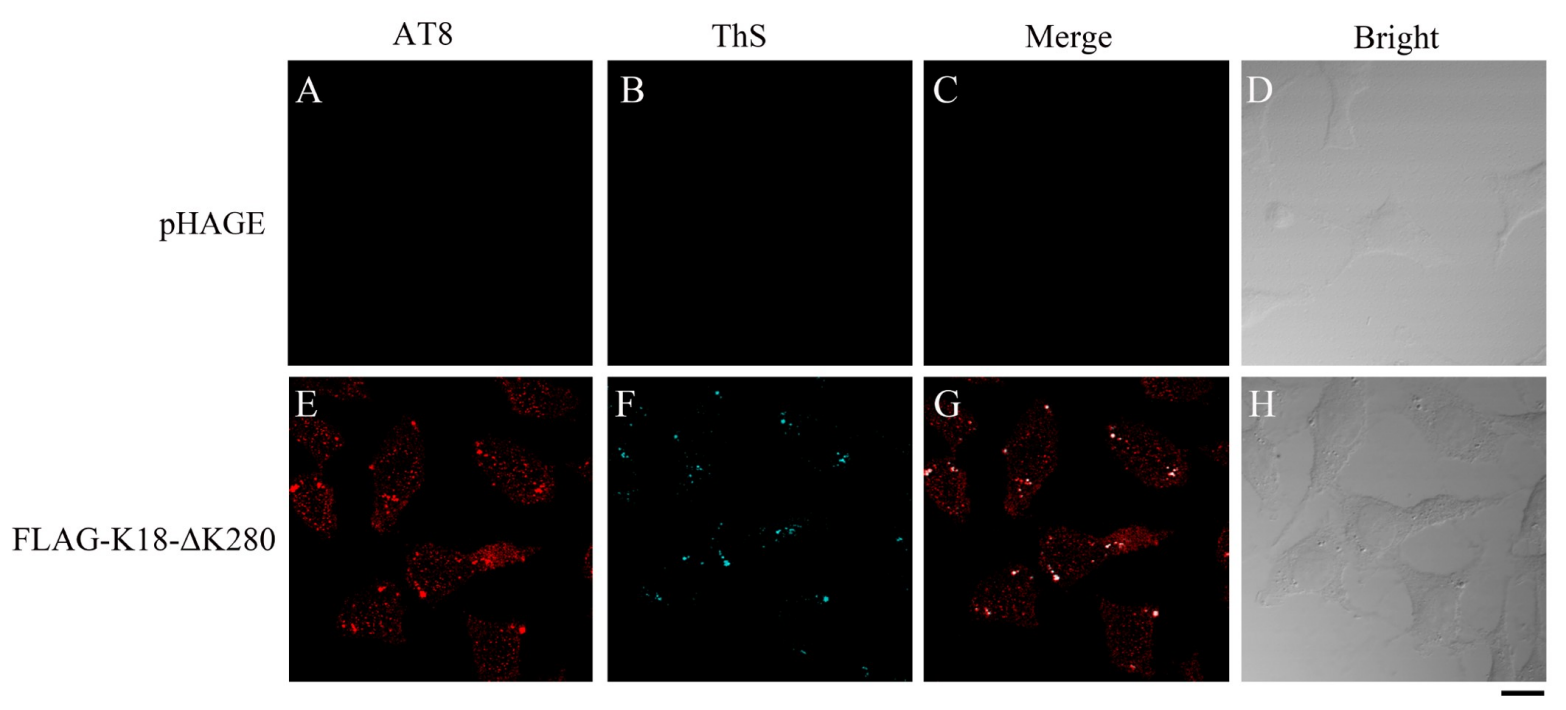
Figure 2 K18-ΔK280 aggregates induce phosphorylation and co-aggregation of endogenous Tau SH-SY5Y cells stably transfected with empty pHAGE plasmid (A–D) or FLAG-tagged K18-ΔK280 (E–H) were fixed with paraformaldehyde, permeabilized with 0.25% Triton X-100, stained with 0.1% ThS (cyan), sequentially coimmunostained with primary monoclonal anti-AT8 antibody (red) and secondary Alexa Fluor 546-conjugated IgG, and visualized by confocal microscopy. Scale bar: 10 μm.

**Figure 3:**
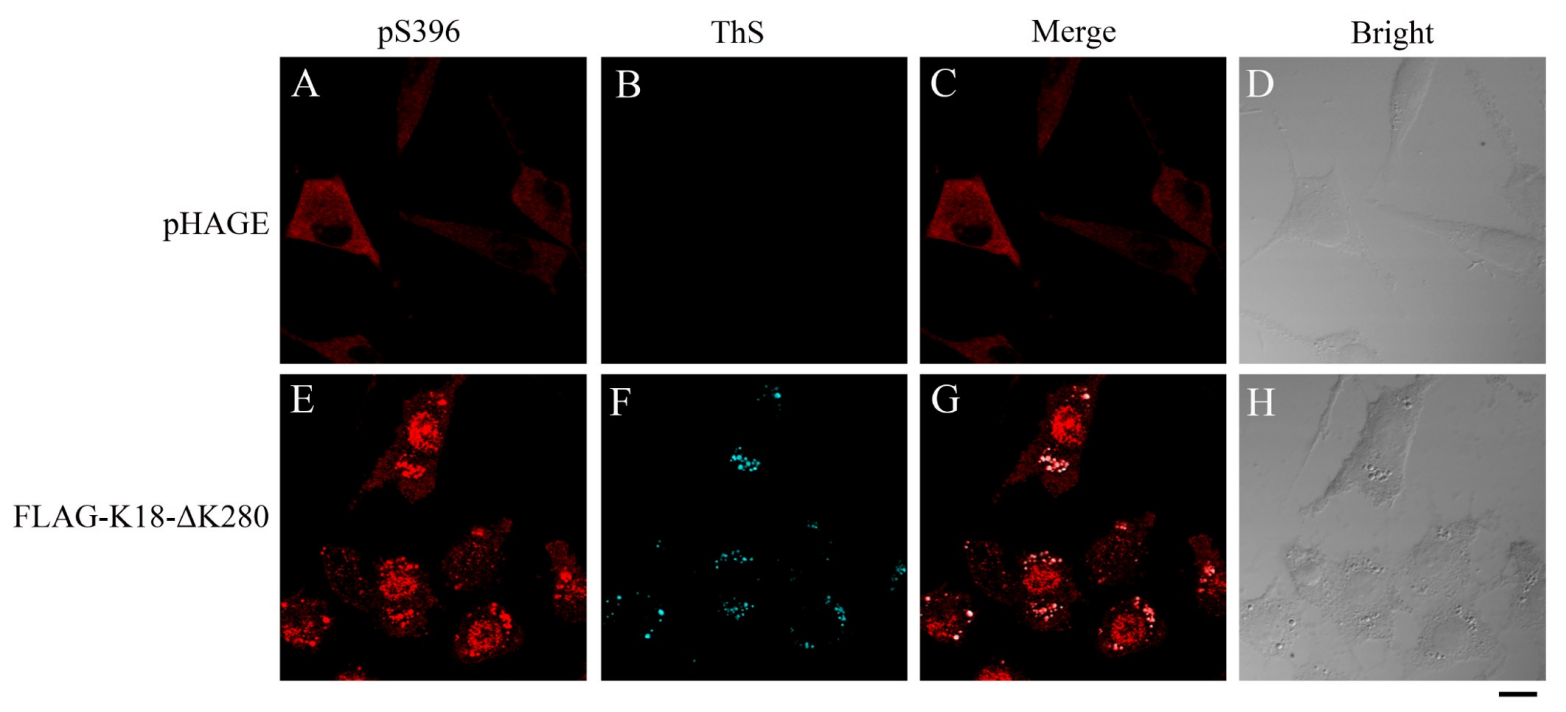
Figure 3 K18-ΔK280 aggregates induce Ser396 phosphorylation and co-aggregation of endogenous Tau SH-SY5Y cells stably transfected with empty pHAGE plasmid (A–D) or FLAG-tagged K18-ΔK280 (E–H) were fixed with paraformaldehyde, permeabilized with 0.25% Triton X-100, stained with 0.1% ThS (cyan), sequentially coimmunostained with primary monoclonal anti-pS396 antibody (red) and secondary Alexa Fluor 546-conjugated IgG, and visualized by confocal microscopy. Scale bar: 10 μm.

A SH-SY5Y cell line stably overexpressing FLAG-tagged K18-ΔK280 was constructed with a lentiviral vector construction system. SH-SY5Y neuroblastoma cells and HEK-293T cells (human renal epithelial cell line) were cultured in Dulbecco’s modified Eagle’s medium supplemented with 10% (v/v) fetal bovine serum (FBS), 100 U/mL penicillin, and 100 U/mL streptomycin in 5% CO _2_ at 37°C. The K18-ΔK280 DNA fragment was cloned and inserted into the lentiviral vector pHAGE-puro via the *Mlu*I and *Bam*HI restriction sites. A lentiviral vector constructed with the CMV promoter was packaged in HEK-293T cells with plasmids and liposome combinations according to the manufacturer’s instructions. After 36 h of transfection, the virus was harvested and filtered. SH-SY5Y cells were infected with the packaged lentivirus with high infection efficiency. Stable cell lines were selected using puromycin.

To determine the expression of K18-ΔK280, stable SH-SY5Y cells were harvested, lysed in Triton lysis buffer (1% Triton X-100, 50 mM Tris, 150 mM NaCl, and protease inhibitors (Roche Diagnostics, Basel, Switzerland) (pH 7.6) on ice for 30 min. The whole-cell lysates were boiled in SDS-PAGE loading buffer, subjected to 13.5% SDS-PAGE and transferred onto polyvinylidene difluoride membranes (Millipore, Billerica, USA). The membranes were incubated with a 1:2000 dilution of monoclonal mouse anti-FLAG antibody (Sigma-Aldrich, St Louis, USA), followed by incubation with goat anti-mouse horseradish peroxidase-conjugated secondary antibody at a dilution of 1:2000. The protein concentrations of the loaded samples were normalized using a BCA protein quantification kit (Beyotime, Shanghai, China). Immunoreactive bands were visualized using enhanced chemiluminescence (BeyoECL Plus; Beyotime). The cells stably transfected with the empty pHAGE vector did not express K18-ΔK280, while the SH-SY5Y cells stably transfected with pHAGE-FLAG-K18-ΔK280 expressed K18-ΔK280 ( Supplementary Figure S1).

K18-ΔK280 expression and localization in SH-SY5Y cells were further investigated by immunofluorescence staining. Stable cells grown on glass cover slips were fixed with 4% paraformaldehyde, permeabilized with 0.25% Triton X-100, blocked with 5% (w/v) BSA, and then sequentially immunostained with an anti-tubulin monoclonal antibody and the corresponding secondary Alexa Fluor 546-conjugated IgG, a primary monoclonal antibody against FLAG and the corresponding secondary Alexa Fluor 488-conjugated IgG, and visualized by confocal microscopy. The samples were observed using an Olympus FluoView FV1000 laser scanning confocal fluorescence microscope (Olympus, Tokyo, Japan) under a 100× oil immersion lens. Tubulin (red) was observed using the Alexa Fluor 559 channel, and K18-ΔK280 (green) was observed using the EGFP channel ( Supplementary Figure S2). K18-ΔK280 expression was not detected in stable cells transfected with the empty pHAGE vector ( Supplementary Figure S2B), while K18-ΔK280 expression was detected in stable cells transfected with pHAGE-FLAG-K18-ΔK280 ( Supplementary Figure S2F). K18-ΔK280 was located mainly in the cytoplasm but did not co-localize with tubulin ( Supplementary Figure S2G). In addition, the cells transfected with FLAG-K18-ΔK280 ( Supplementary Figure S2E‒H) or pHAGE ( Supplementary Figure S2A‒D) presented different cell shapes, which was possibly caused by the overexpression of K18-ΔK280.

To detect the spontaneous fibrillization of K18-ΔK280 in SH-SY5Y cells, stable SH-SY5Y cells grown on glass cover slips were fixed, permeabilized, blocked, stained with 0.1% ThS, sequentially coimmunostained with a primary monoclonal anti-FLAG antibody and then with an Alexa Fluor-546-linked secondary antibody, and finally visualized by confocal microscopy. ThS (cyan) was observed in the ECFP channel; Alexa Fluor 546 (red) was observed in the Alexa Fluor 559 channel. As shown in Figure 1, no ThS-positive aggregates were formed ( Figure 1B) in cells stably transfected with the empty pHAGE vector ( Figure 1A–D). In contrast, ThS-stainable aggregates ( Figure 1F) were formed in stable K18-ΔK280 cells ( Figure 1E–H), and the aggregates were mainly located in the cytoplasm ( Figure 1G). It should be mentioned that we do not know why the FLAG-antibody staining did not show amyloid dots ( Figure 1E), as expected, although K18-ΔK280 is an aggregation-prone fragment of Tau.

Based on the spontaneous aggregation of K18-ΔK280, two phosphorylation-specific antibodies, AT8 and pS396, which recognize the phospho-Ser202/Thr205 and phospho-Ser396 amino acids, respectively, of the full-length human Tau protein, which is located outside the amino acid sequence of K18-ΔK280, were used to detect the phosphorylation and aggregation status of the cellular endogenous Tau protein. Stable SH-SY5Y cells grown on glass cover slips were fixed, permeabilized, blocked, stained with 0.1% ThS, sequentially coimmunostained with primary monoclonal antibodies against AT8 or pS396, incubated with the corresponding Alexa Fluor-546-linked secondary antibodies, and finally visualized by confocal microscopy. ThS (cyan) was observed in the ECFP channel; Alexa Fluor 546 (red) was observed in the Alexa Fluor 559 channel.

The AT8 antibody can detect the endogenous Tau protein ( Figure 2), similar to the pS396 antibody ( Figure 3), and the two antibodies against endogenous Tau can detect the Tau protein that colocalizes with the amyloid-like aggregates detected by ThS, showing that the K18-ΔK280 aggregates entrap aggregation of the endogenous Tau protein ( Figures 2 and 3). No ThS-positive aggregates were formed in stable pHAGE cells ( Figures 2B and 3B), whereas ThS-positive aggregates were detected in K18-ΔK280 stable cells ( Figures 2F and 3F), suggesting that the protein formed aggregates in the cells. The phosphorylation of Ser202 and Thr205 was detected only in cells stably expressing K18-ΔK280 ( Figure 2E) but not in cells stably transfected with the empty pHAGE vector ( Figure 2A), suggesting that the expression and aggregation of K18-ΔK280 induce the phosphorylation of Ser202 and Thr205 in the endogenous Tau protein. The phosphorylation of Ser396 in K18-ΔK280 stable cells was greater than that in stable pHAGE cells ( Figure 3A,E). Moreover, the partially phosphorylated proteins showed excellent colocalization with ThS-stained aggregates formed by K18-ΔK280 ( Figures 2G and 3G). Together, these results indicated that K18-ΔK280 aggregation promotes the phosphorylation of endogenous Tau at Ser202/Thr205 and Ser396, resulting in the co-aggregation of endogenous Tau.

K18-ΔK280 is an aggregation-prone truncated core fragment of Tau (Tau _244‒372_, K18). The aggregates formed by this mutant may induce the formation of aggregates of the endogenous Tau protein via a co-aggregation mechanism [ 11– 13] . In this work, we generated a cell-based aggregation model for Tau by overexpressing K18-ΔK280 in SH-SY5Y cells. K18-ΔK280 aggregates act as seeds, thereby accelerating the aggregation of endogenous Tau. The cellular model for Tau aggregation established in this study does not require preprepared seeds, thereby avoiding the potential cell toxicity and low induction efficiency caused by poor transmembrane permeability of seeds. By using phosphorylation-specific antibodies, it is also possible to simultaneously study the effect of Tau aggregation on abnormal Tau phosphorylation. The development of anti-AD drugs targeting the Tau protein at the cellular level is valuable for studying Tau aggregation, posttranslational modifications, and pathological mechanisms.

## Supporting information

23542Supplementary_Figures

## Supplementary Data

Supplementary data is available at *Acta Biochimica et Biophysica Sinica* online.
