# Supplementary material for: Construction of a cell-based aggregation and seeding model for the Tau protein: A cell-based aggregation and seeding model for the Tau protein
Source: Acta Biochim Biophys Sin (Shanghai). 2024 Apr 29;56(7):1085–8. doi: 10.3724/abbs.2024057 (PMC11322869; doi:10.3724/abbs.2024057)
Supplement: 23542Supplementary_Figures [file 23542Supplementary_Figures.pdf]

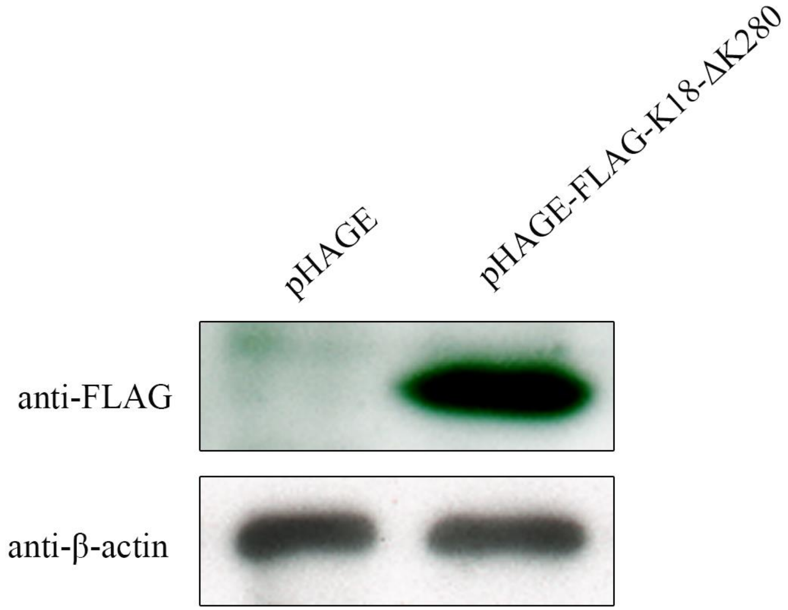

Supplementary Figure S1. Expression of K18-ΔK280 in stable SH-SY5Y cells The expression of K18-ΔK280 was detected in SH-SY5Y cells stably transfected with pHAGE-FLAG-K18-ΔK280 by western blot analysis, compared with those stably transfected with empty pHAGE vector. Only stable K18-Δ280 cell line showed protein expression.

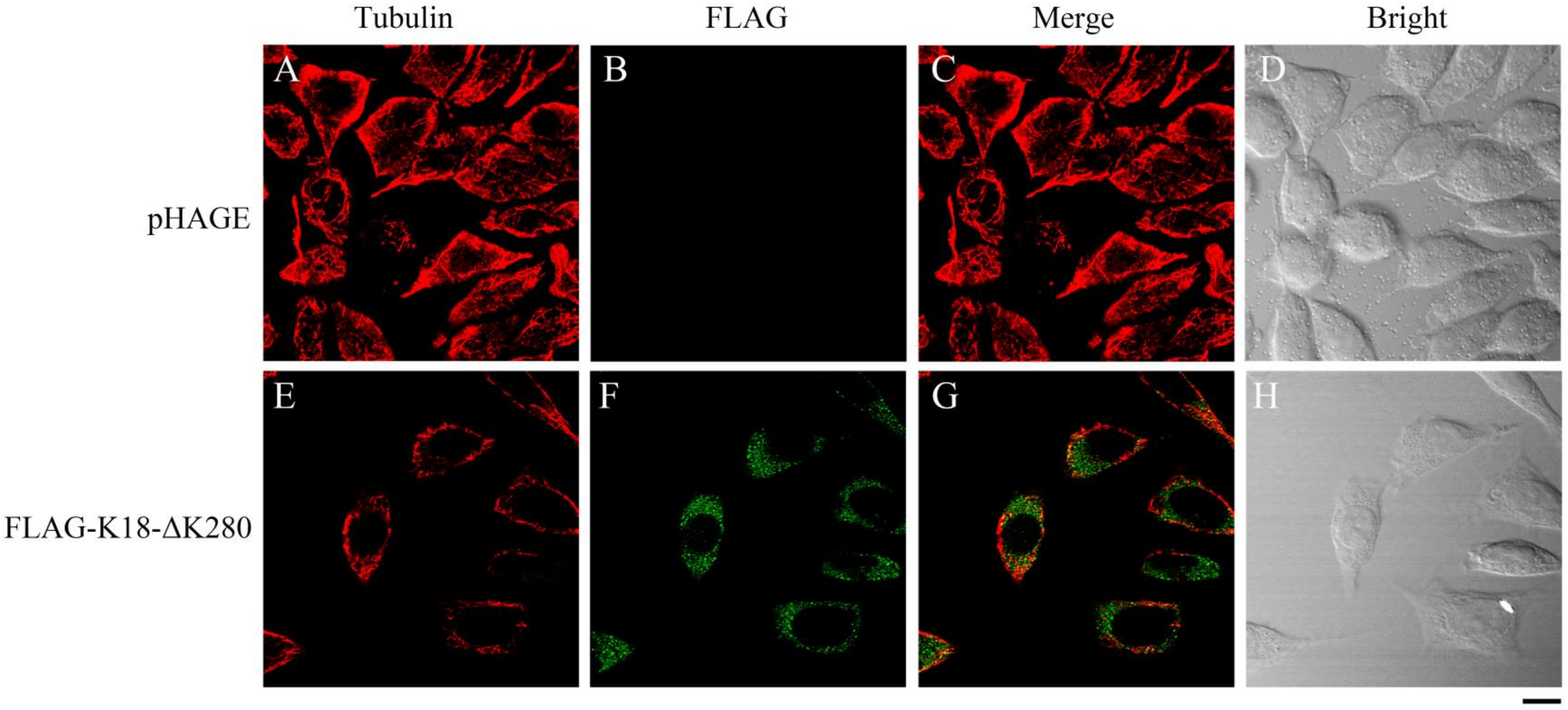

Supplementary Figure S2. Expression and localization of FLAG-tagged K18-ΔK280 in stable SH-SY5Y cells detected by immunofluorescence SH-SY5Y cells stably transfected with empty pHAGE vector (A–D) or FLAG-tagged K18-ΔK280 (E–H) were fixed with paraformaldehyde, permeabilized with 0.25% Triton X-100, sequentially immunostained with anti-tubulin monoclonal antibody and the corresponding secondary Alexa Fluo-546 conjugated IgG, primary monoclonal antibody anti-FLAG and the corresponding secondary Alexa

Fluor-488 conjugated IgG, and visualized by confocal microscopy. Scale bar: 10 μm.
